# Supplementary figures and images for: 3D printing of different fibres towards HA/PCL scaffolding induces macrophage polarization and promotes osteogenic differentiation of BMSCs
Source: PLoS One. 2025 Jan 13;20(1):e0314150. doi: 10.1371/journal.pone.0314150 (PMC11729943; doi:10.1371/journal.pone.0314150)

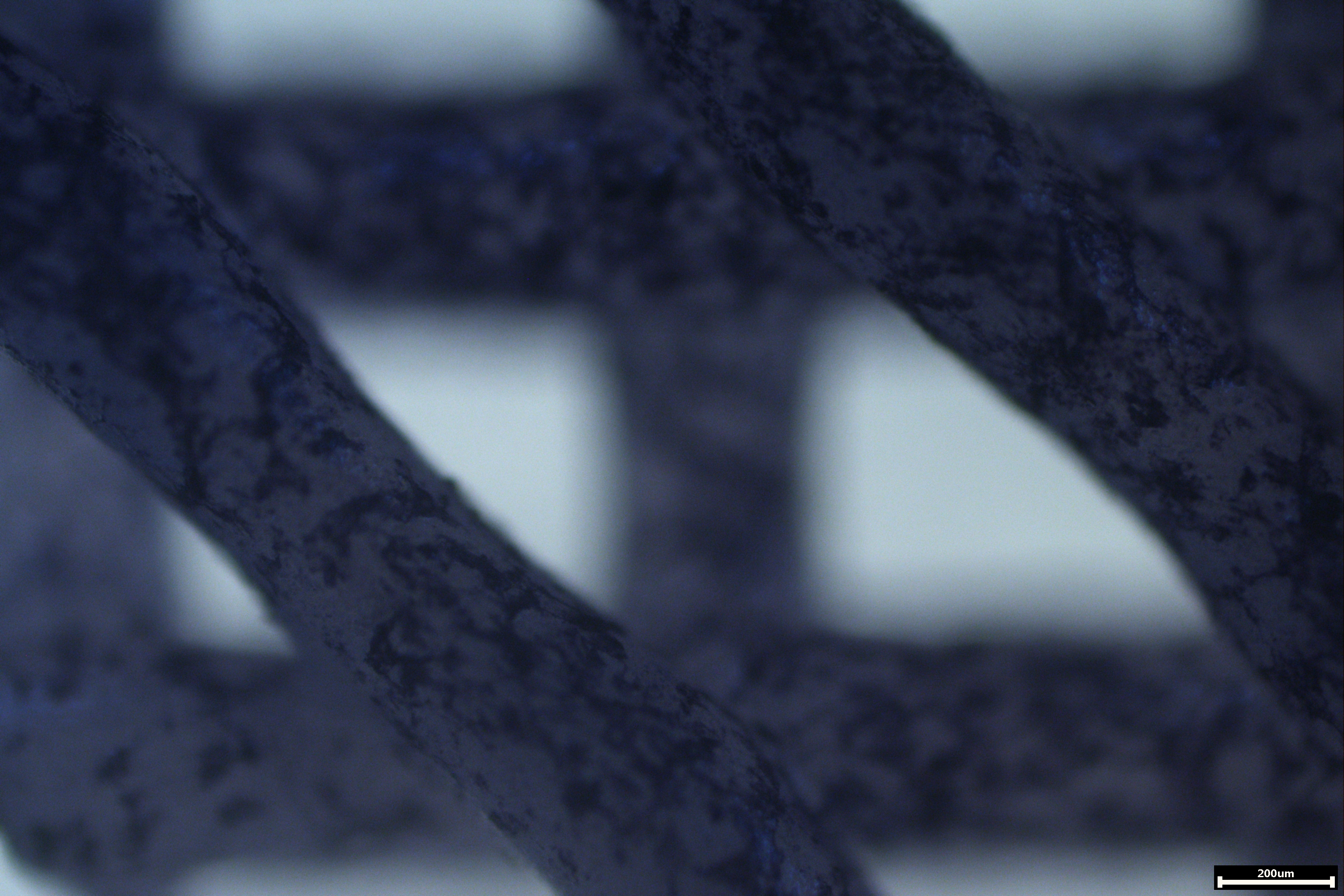

Supplement: S1 File — (ZIP) [file pone.0314150.s001.zip › ALP staining/90-135.jpg]

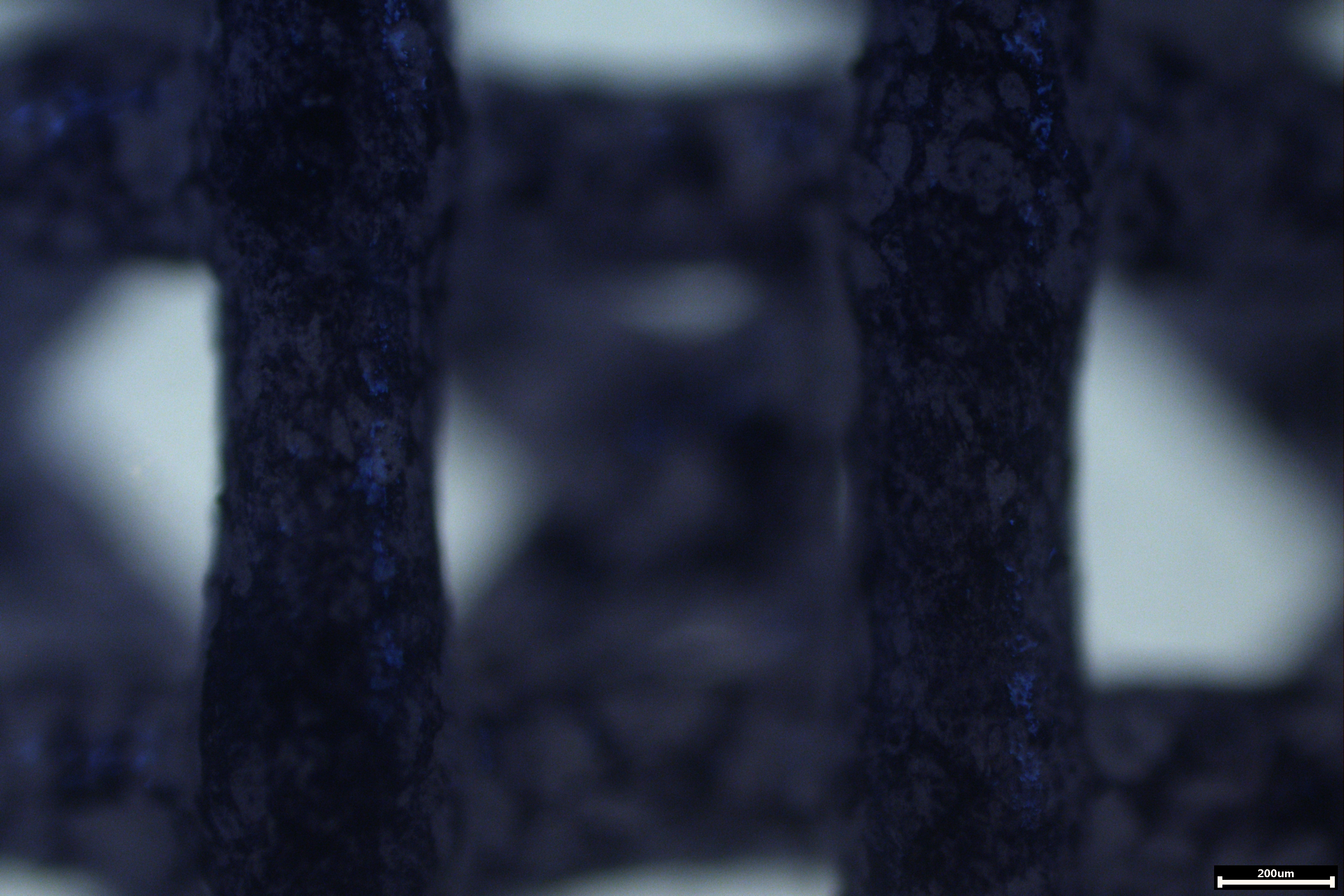

Supplement: S1 File — (ZIP) [file pone.0314150.s001.zip › ALP staining/90-45.jpg]

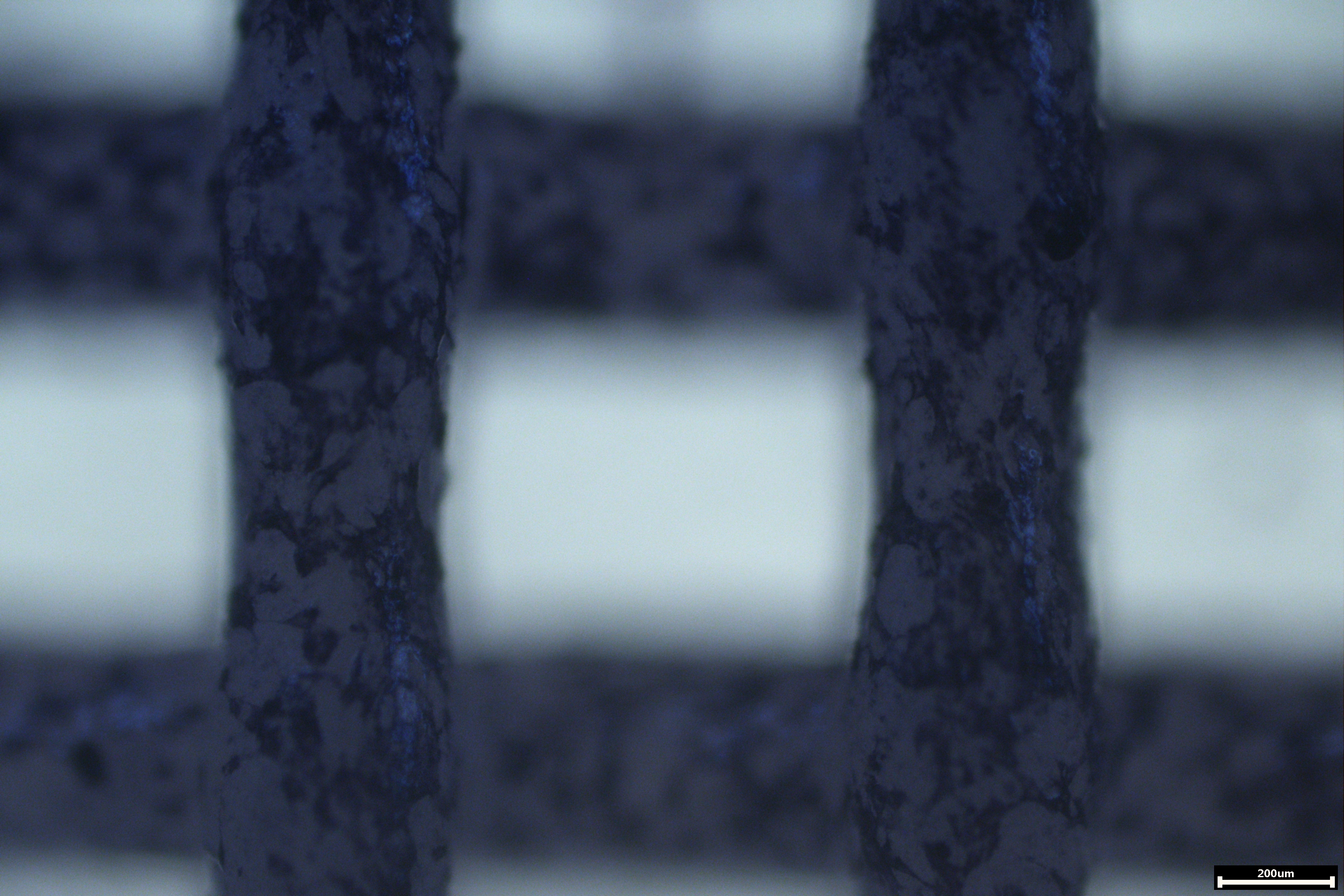

Supplement: S1 File — (ZIP) [file pone.0314150.s001.zip › ALP staining/90.jpg]

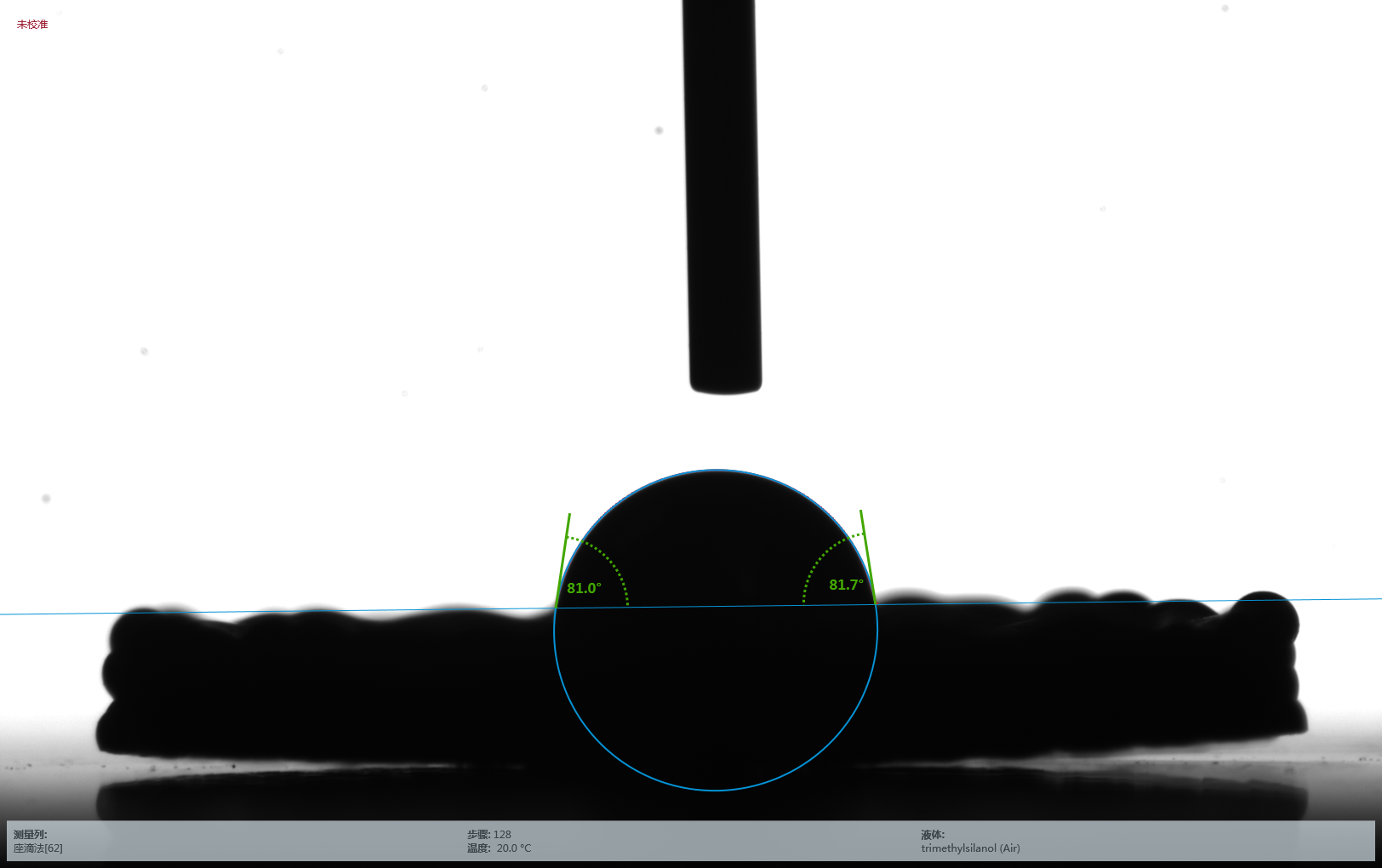

Supplement: S1 File — (ZIP) [file pone.0314150.s001.zip › Contact angle/90-135.png]

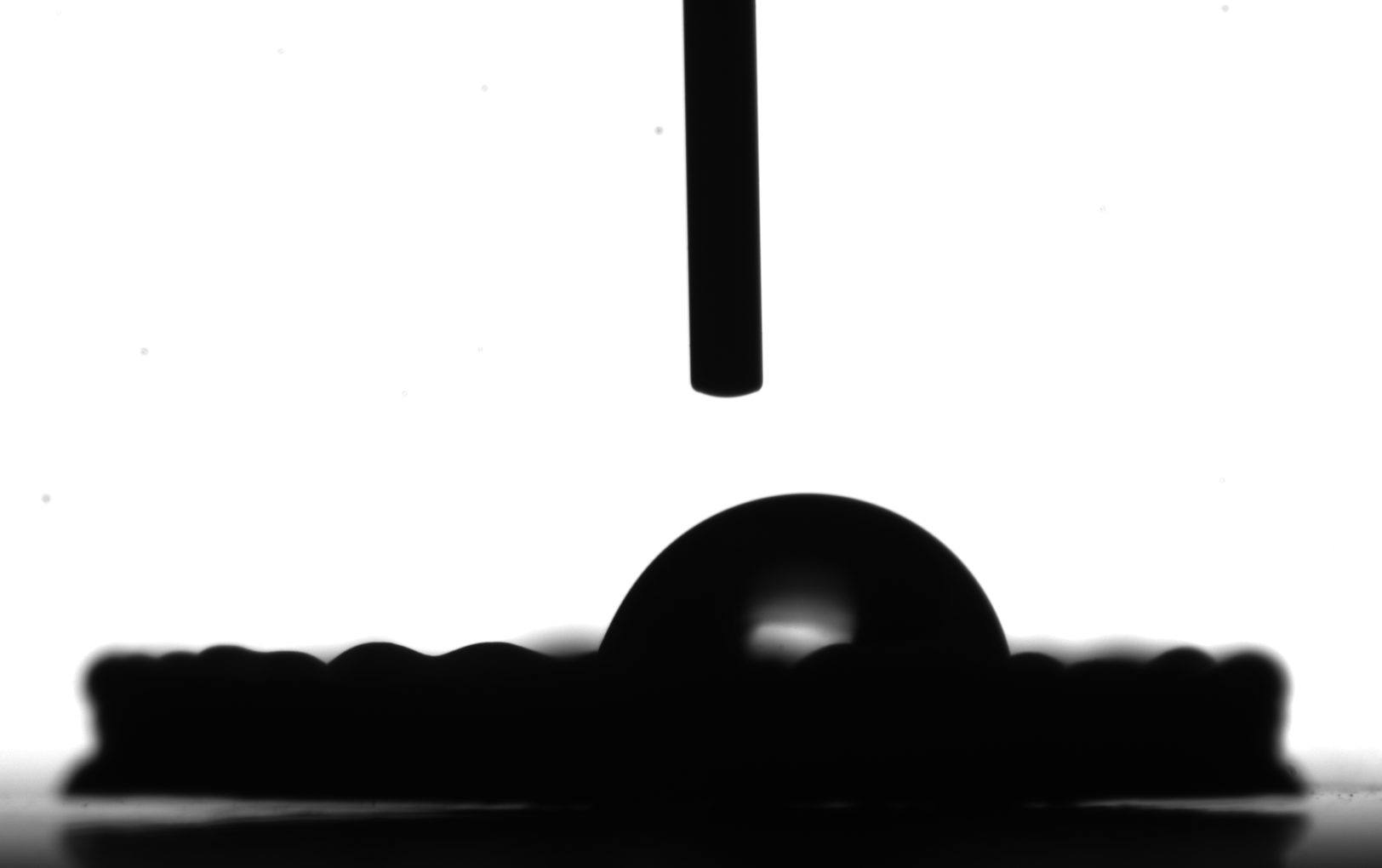

Supplement: S1 File — (ZIP) [file pone.0314150.s001.zip › Contact angle/90-45.png]

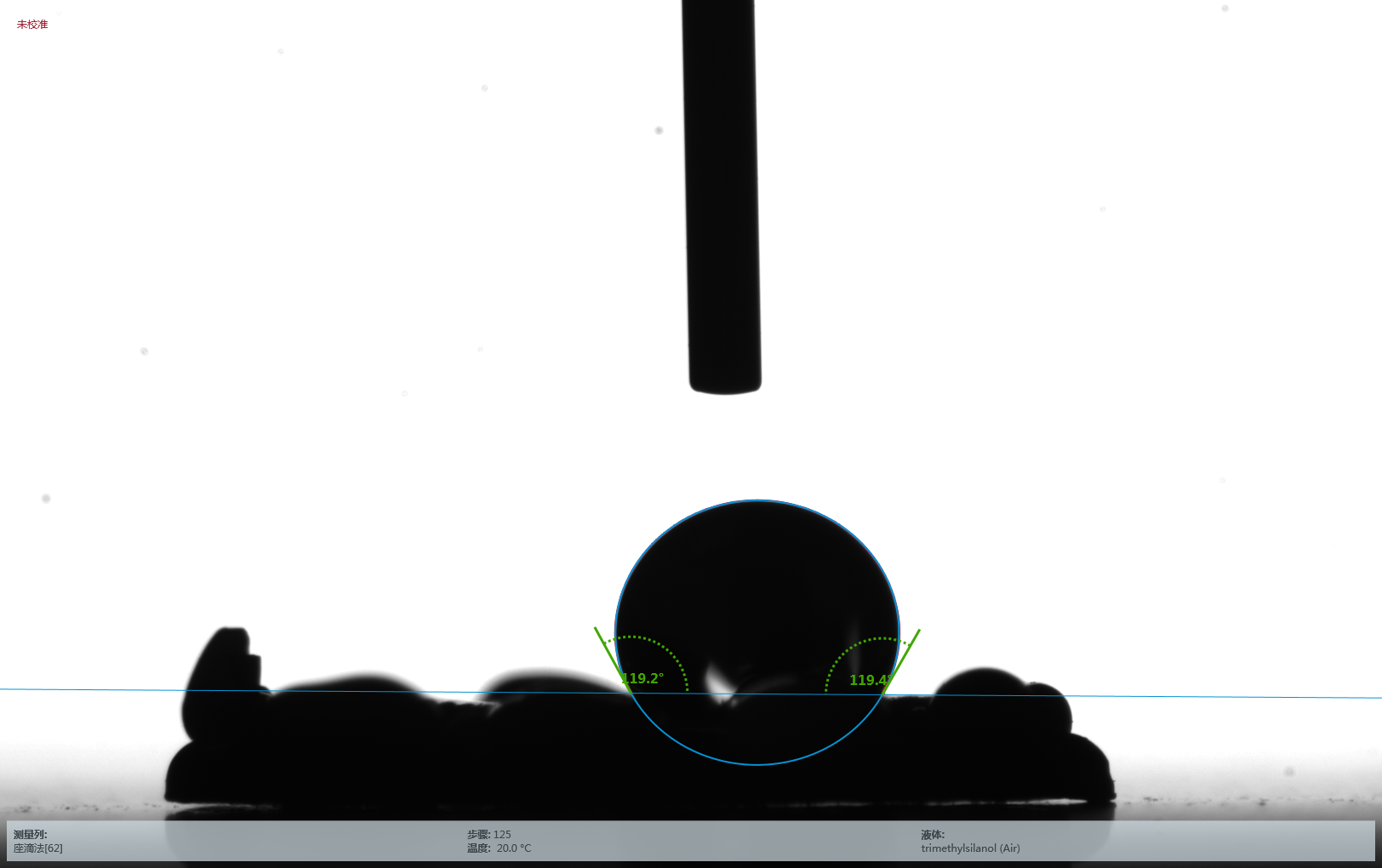

Supplement: S1 File — (ZIP) [file pone.0314150.s001.zip › Contact angle/90.png]

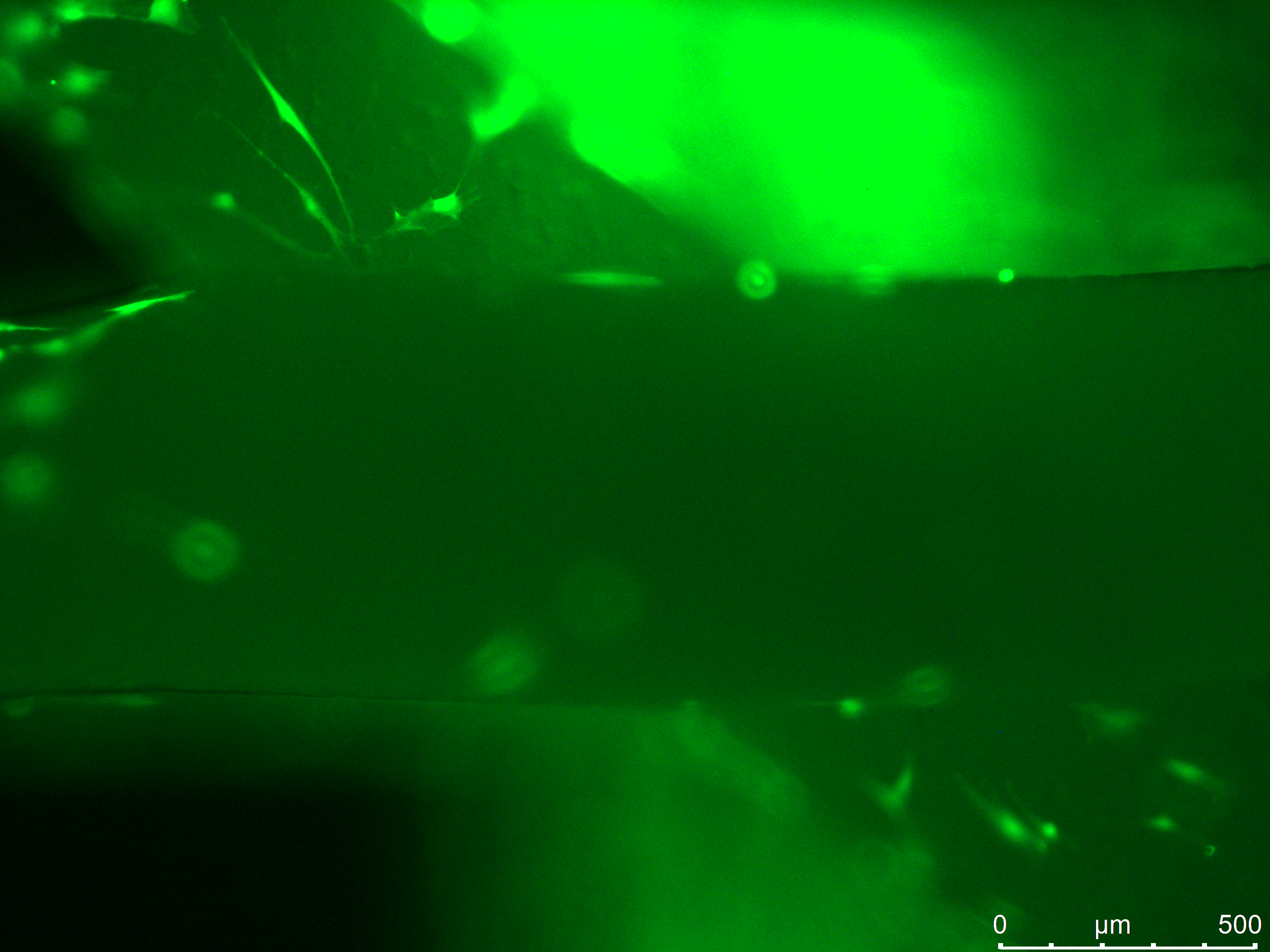

Supplement: S1 File — (ZIP) [file pone.0314150.s001.zip › Live-die/90-135.tif]

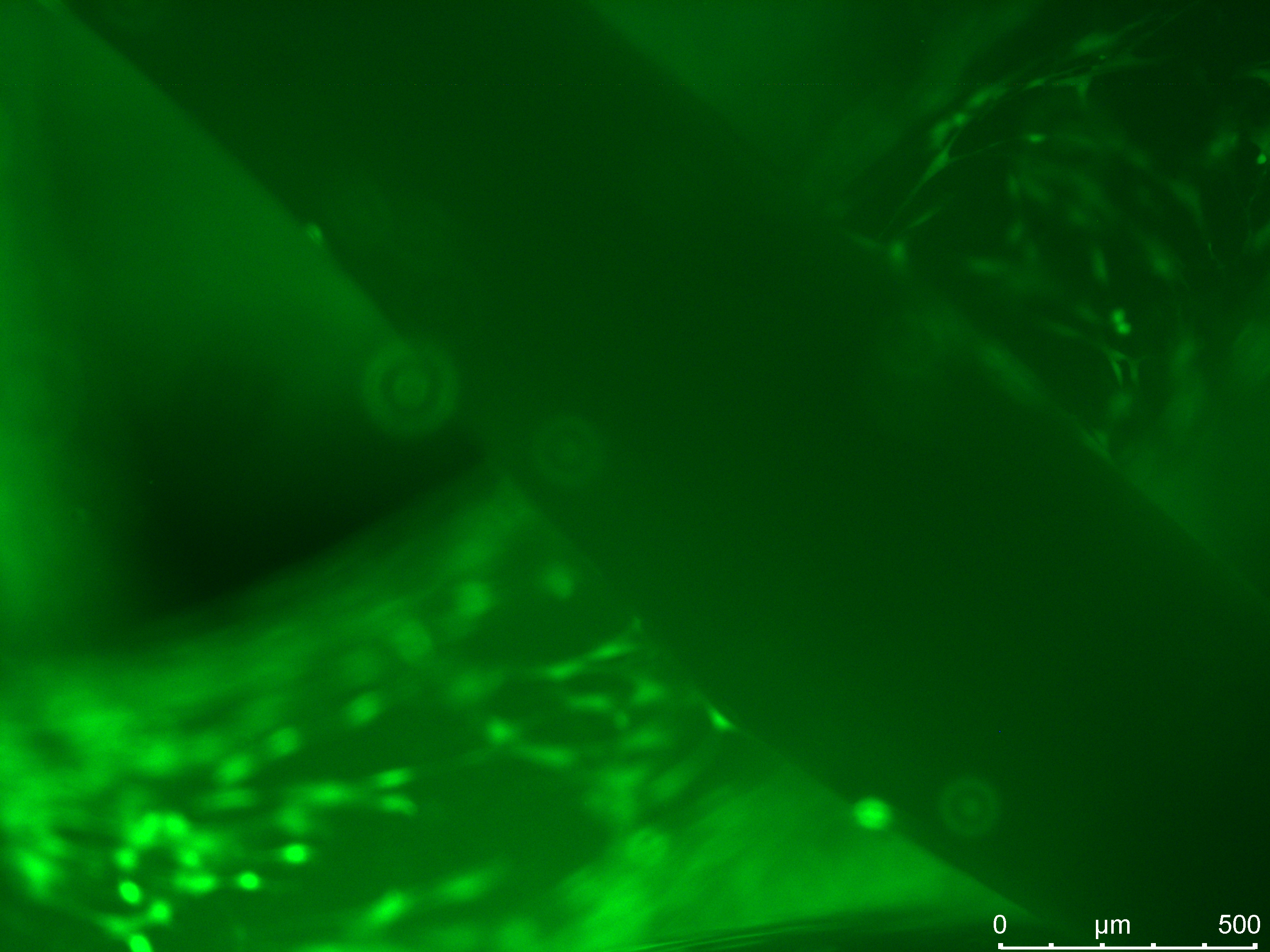

Supplement: S1 File — (ZIP) [file pone.0314150.s001.zip › Live-die/90-45.tif]

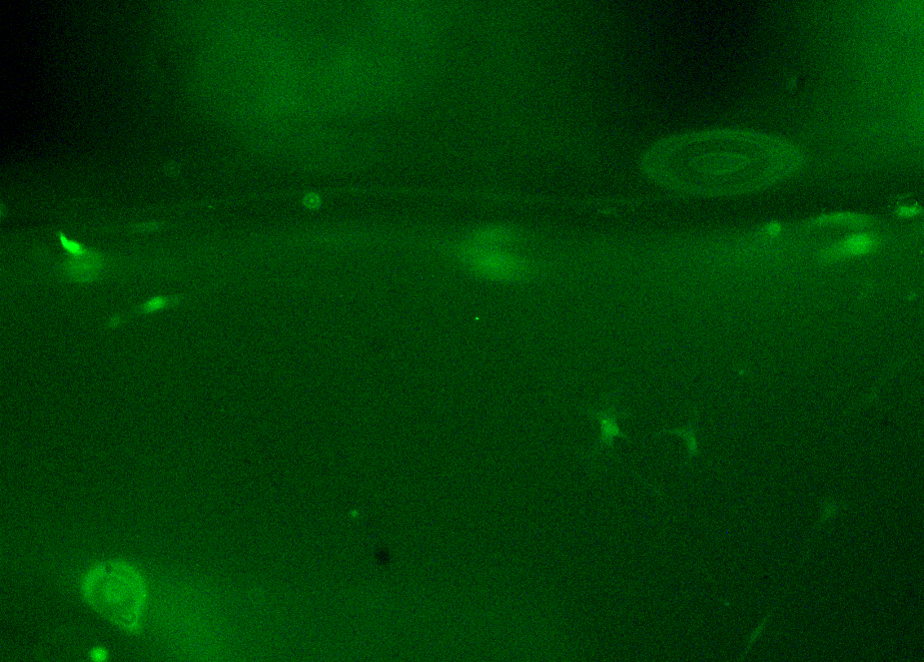

Supplement: S1 File — (ZIP) [file pone.0314150.s001.zip › Live-die/90.tif]

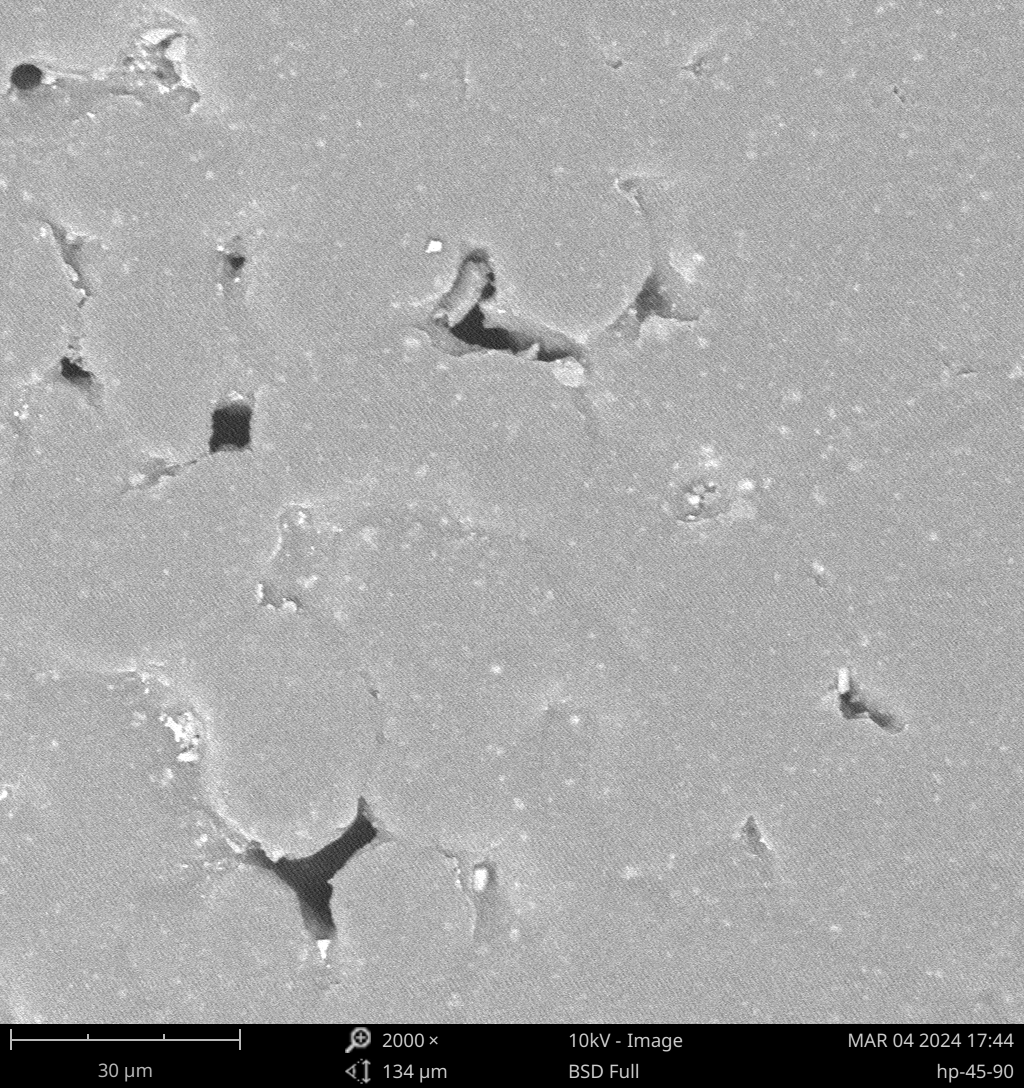

Supplement: S1 File — (ZIP) [file pone.0314150.s001.zip › SEM/45-90/hp-45-900006.tiff]

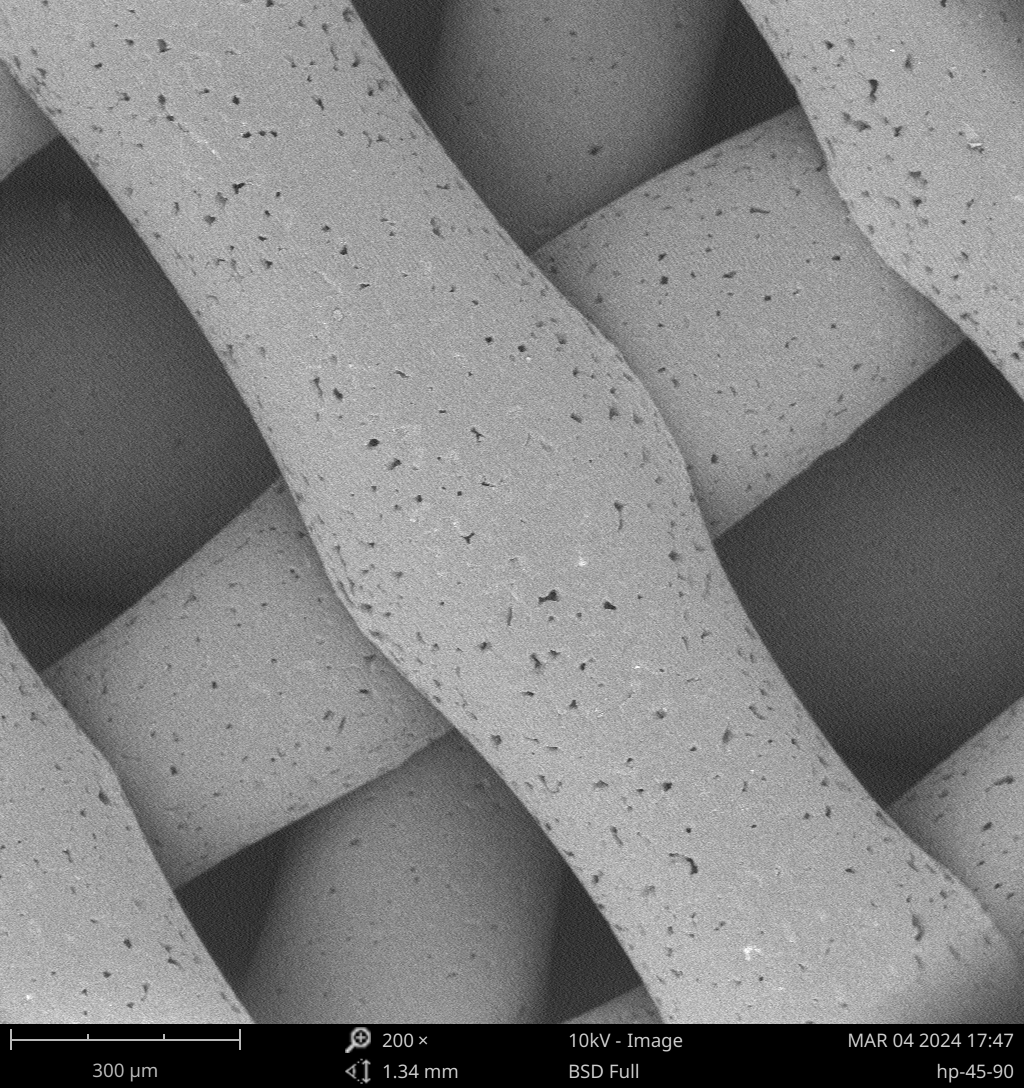

Supplement: S1 File — (ZIP) [file pone.0314150.s001.zip › SEM/45-90/hp-45-900009.tiff]

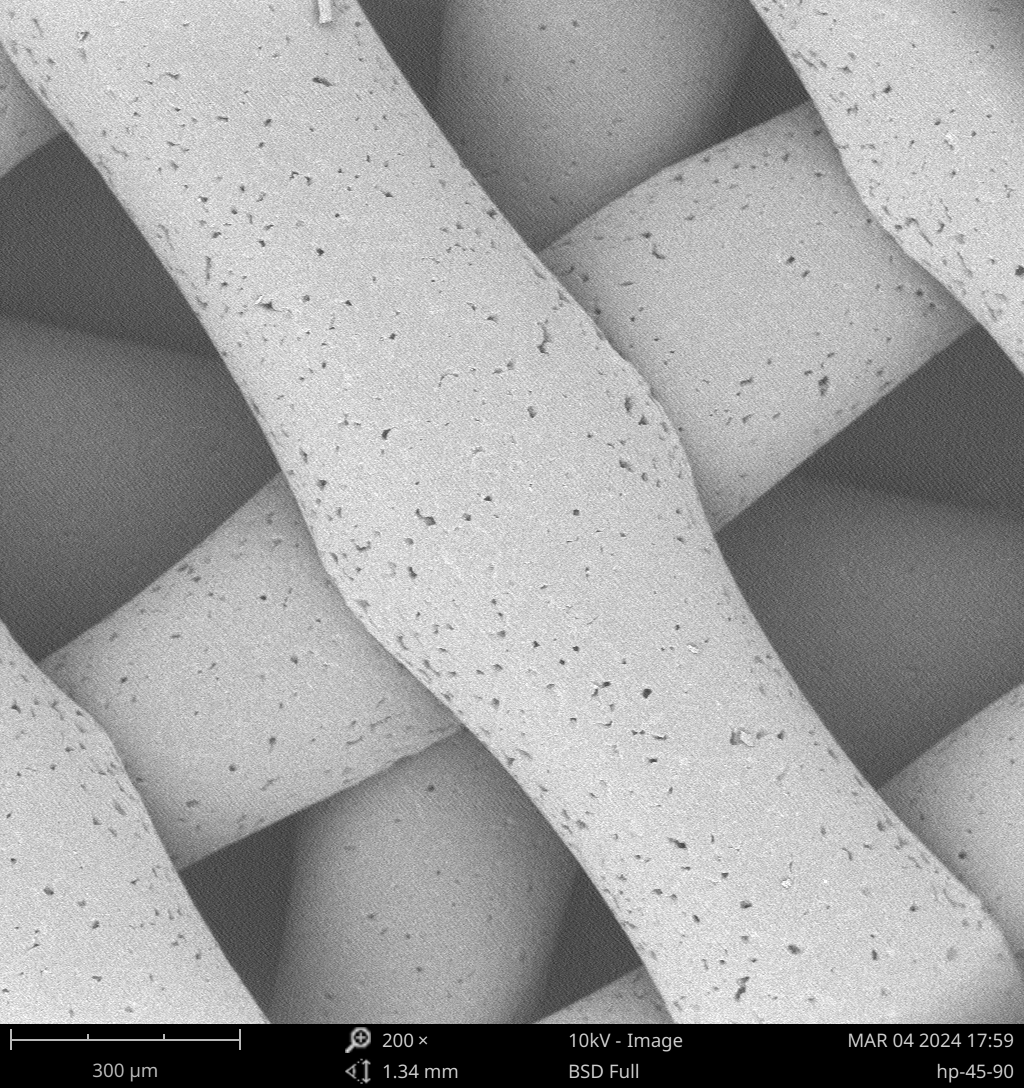

Supplement: S1 File — (ZIP) [file pone.0314150.s001.zip › SEM/45-90/hp-45-900018.tiff]

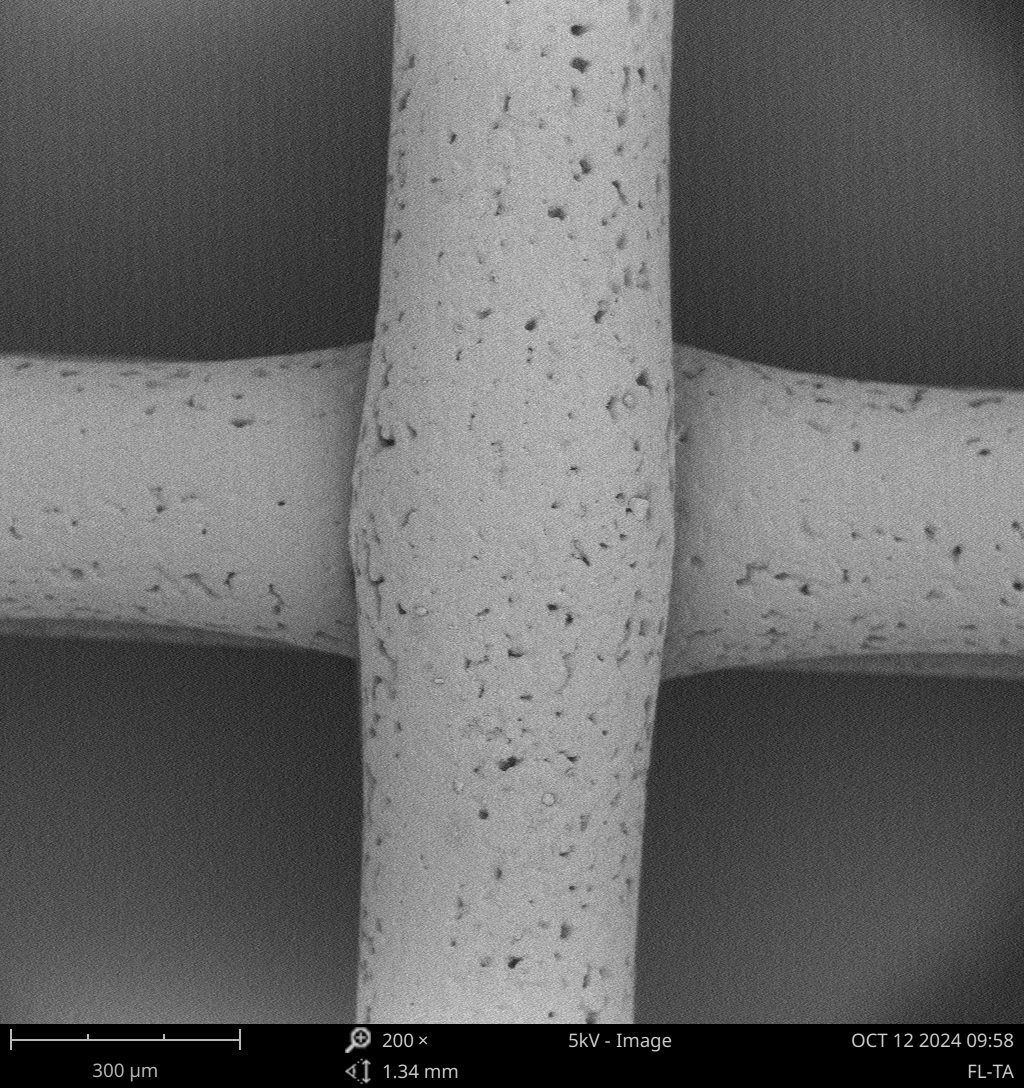

Supplement: S1 File — (ZIP) [file pone.0314150.s001.zip › SEM/90/FL-TA0001.tiff]

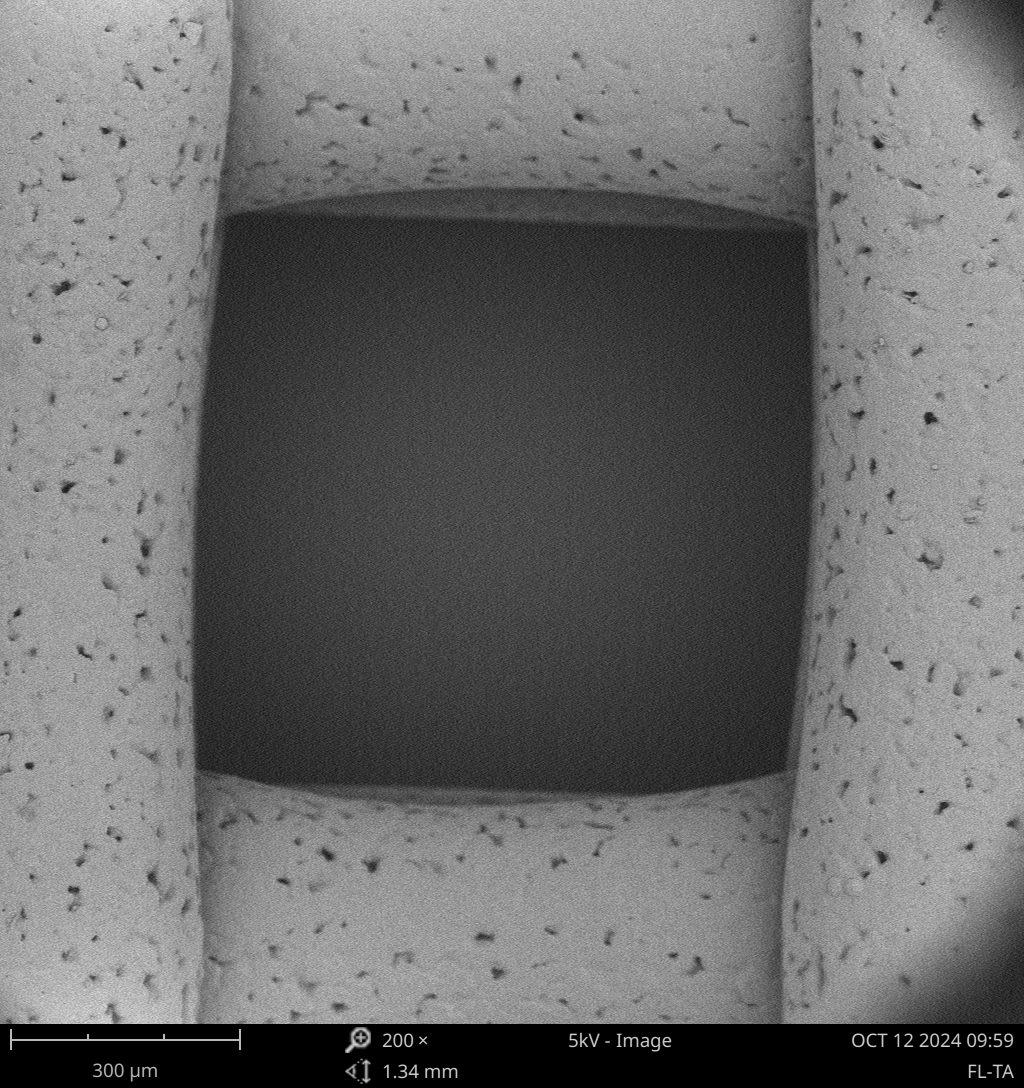

Supplement: S1 File — (ZIP) [file pone.0314150.s001.zip › SEM/90/FL-TA0002.tiff]

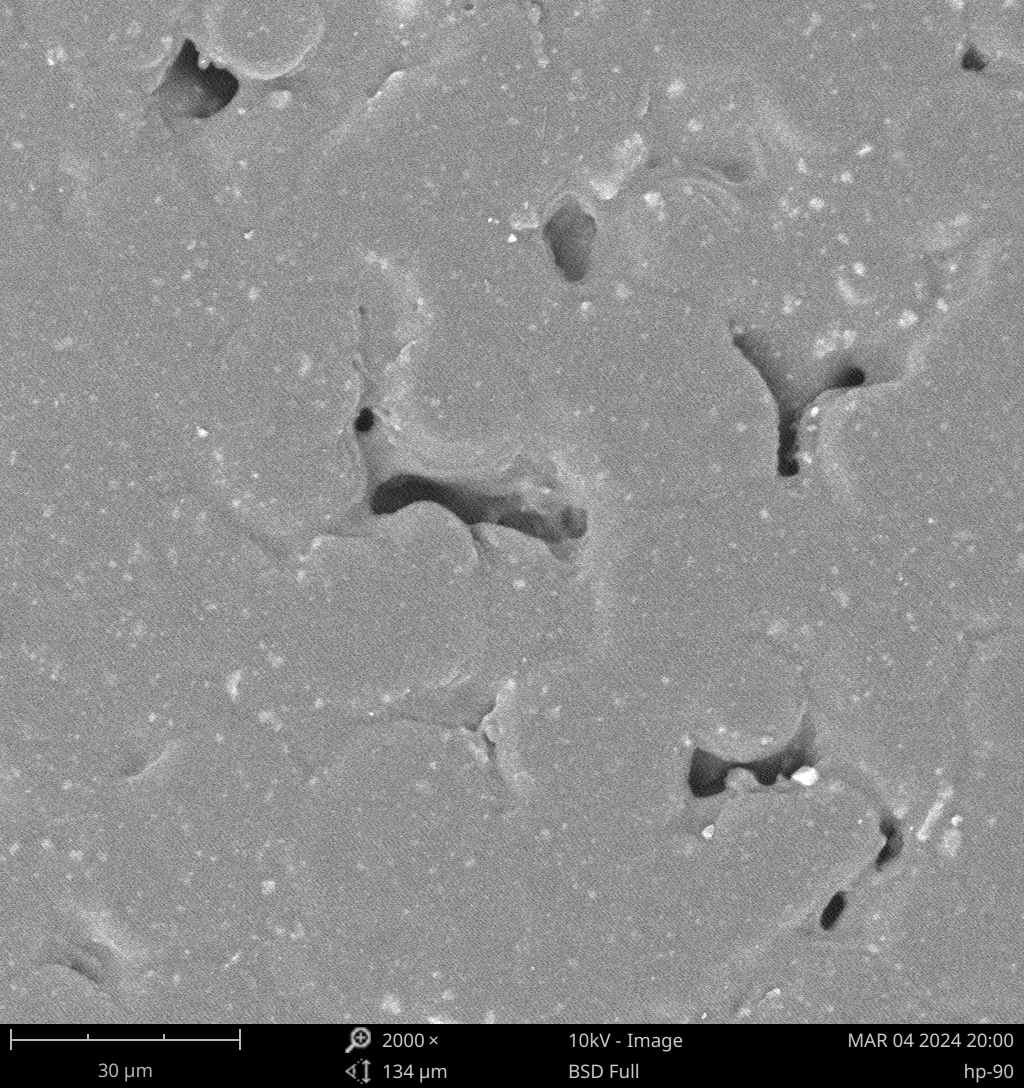

Supplement: S1 File — (ZIP) [file pone.0314150.s001.zip › SEM/90/hp-900013.tiff]

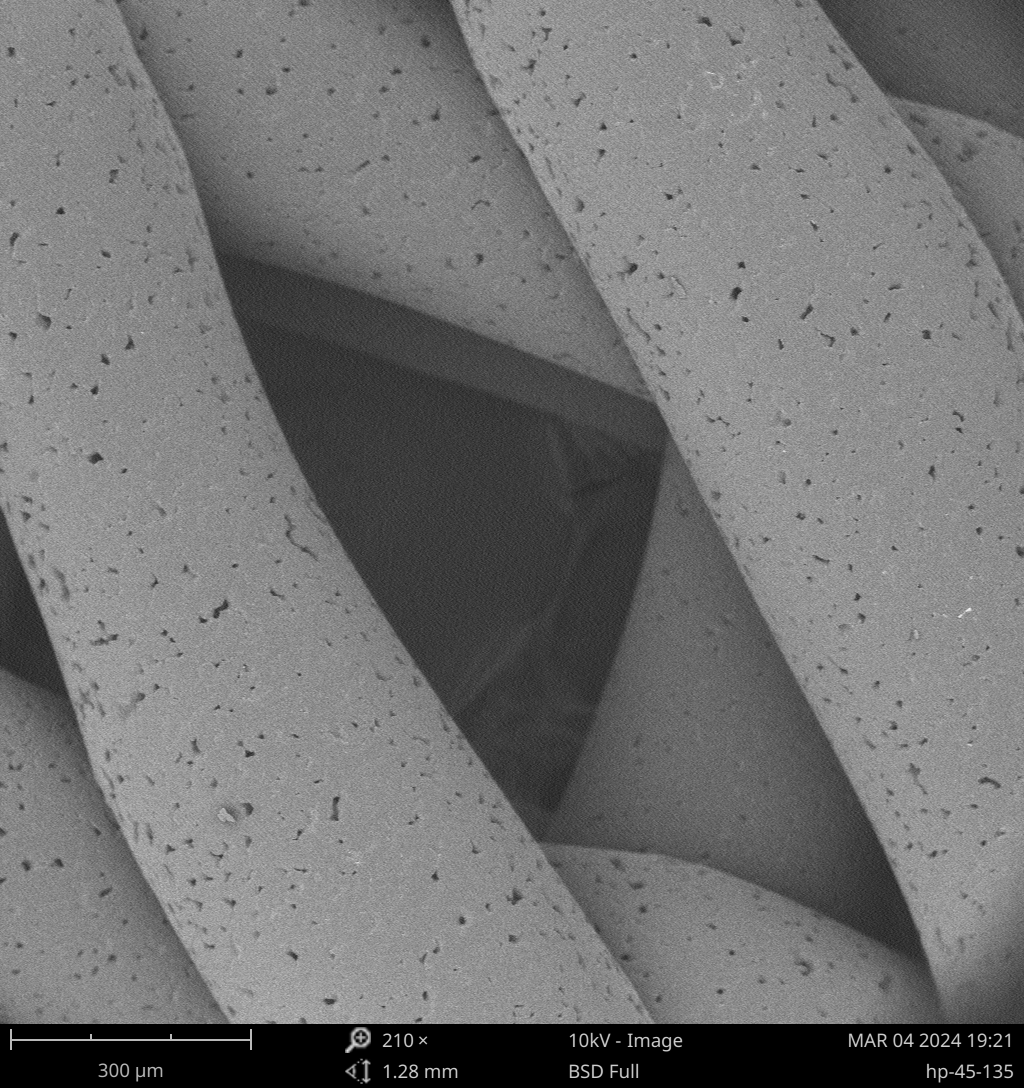

Supplement: S1 File — (ZIP) [file pone.0314150.s001.zip › SEM/90-135/hp-45-1350018.tiff]

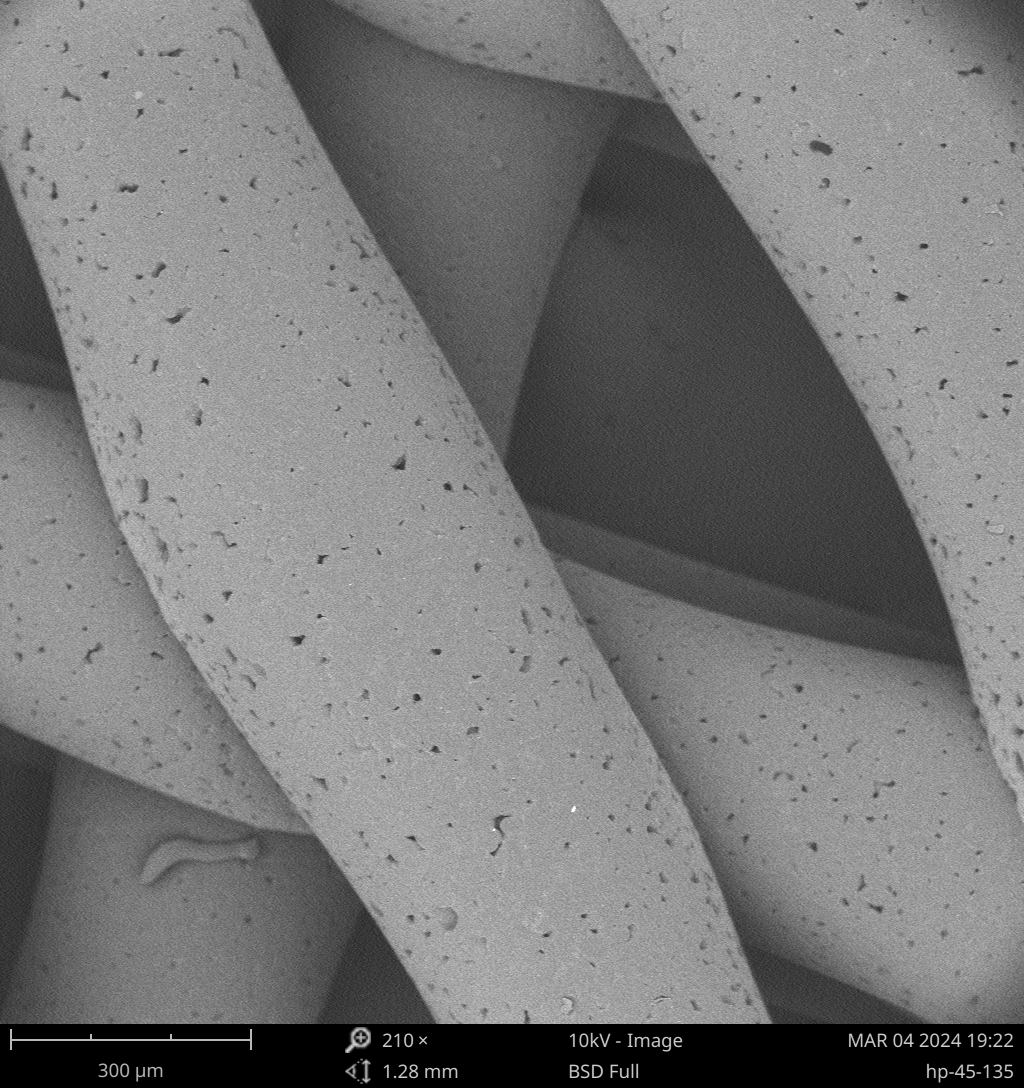

Supplement: S1 File — (ZIP) [file pone.0314150.s001.zip › SEM/90-135/hp-45-1350019.tiff]

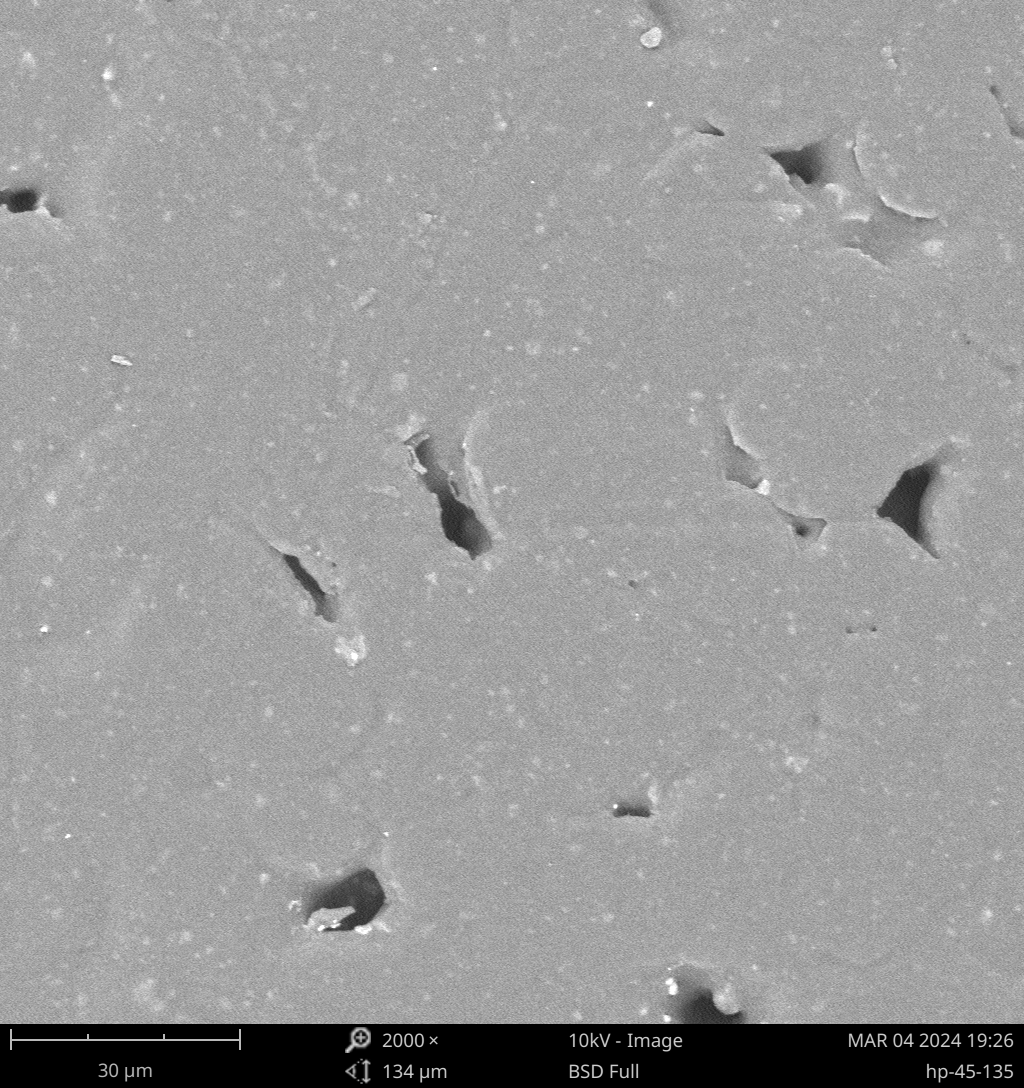

Supplement: S1 File — (ZIP) [file pone.0314150.s001.zip › SEM/90-135/hp-45-1350023.tiff]
