# Supplementary material for: 3D printing of different fibres towards HA/PCL scaffolding induces macrophage polarization and promotes osteogenic differentiation of BMSCs
Source: PLoS One. 2025 Jan 13;20(1):e0314150. doi: 10.1371/journal.pone.0314150 (PMC11729943; doi:10.1371/journal.pone.0314150)
Supplement: Attachment — Submitted filename: Response to reviewers.doc [file pone.0314150.s002.doc]

Reviewer #1: The manuscript is well-written and clearly communicates the study's objectives and findings. It provides a comprehensive overview of the research on 3D-printed HA/PCL scaffolds, highlighting key aspects such as fibre orientation, osteogenic induction, and macrophage polarization. The novelty lies in exploring different fibre orientations and their effects on scaffold properties and biological responses, which offers valuable insights into the field. This work could be considered for publication after addressing the following comments:
- Section 2.1: Please provide more information about the HA powder used (e.g., particle size, purity, etc.).

Dear reviewer, we have added this section.

Since fibre orientation could significantly affect the mechanical and physical properties of the fabricated scaffolds via the 3D printing method, please present the results shown in Fig. 1 in at least two or three separate figures for better illustration.

Dear reviewer, we have added accordingly.

Section 2.2: HA powder does not dissolve in dichloromethane; it is dispersed. Please revise accordingly.

Dear reviewer, we have revised it.

Section 2.2: What does “mm I.D. printing needle” mean?

Dear reviewer, this was a writing error on our part and we have revised this part of the description.

Gene expression is typically evaluated over one month, but the authors studied it for only 14 days. What is the reason for this shorter duration?

Dear reviewer, the early regulation of BMSCs by scaffolding is more significant, and the significance of the difference between groups is weakened if the time is too long to account for. The current mainstream studies on osteogenic differentiation of BMSCs are also at the time points of day 7 and 14[1, 2].

[1] Y. Miao, X. Liu, J. Luo, Q. Yang, Y. Chen, Y. Wang, Double-Network DNA Macroporous Hydrogel Enables Aptamer-Directed Cell Recruitment to Accelerate Bone Healing, Advanced Science 11(1) (2024).

[2] X. Ren, Q. Zhou, M. Oberoi, R. Caprini, N. Dahan, B. Harley, J. Lee, Physisorption of Recombinant Osteoprotegerin on Nanoparticulate Mineralized Collagen Scaffolds Reduces Osteoclast Resorption Activity without Affecting Osteogenesis, Journal of Bone and Mineral Research 37 (2022) 24-24.

Reviewer #2: I am a bit confused after reading the paper. The biological part seems good but the characterization of the material is very ambiguous. In this form, I cannot recommend the publication. In my opinion, the 3D printed scaffold have to be better characterized and after this more correlations to be done with the biological data. Some of the most important idea I noticed during the lecture of the manuscript are listed bellow

HA was dispersed and not dissolved in DCM

Dear reviewer, thank you for the reminder, we have fixed it.

It is strange at all that the angle between the strands is considered but the distance between these are not considered. Size is critical in bone tissue engineering and cells need pores of 50 -150 um to be able to get inside them and to assure a proper ingrowth. Even if some references are done related to pore size, this part is not at all described. Also, the porosity is greater than 70% but I am not sure if it is determined experimentally of just estimated based on the printing model.

Dear reviewer, it is us who overlooked this part. When we wrote the programming file, we designed the fiber size of 500 um, and the spacing of the fibers is 900 um. 0-90 is the fibers intersecting at 90^o^, and the four fibers constitute an aperture with an area of 8.1×10^5^ um^2^. 0-90-135 is the addition of a 135^o^ fiber to 0-90, which divides the original aperture into two equilateral right triangles with a base side of 650 um, and the theoretical aperture area of 0-90-135 is 4.225×10^5^ um^2^. The theoretical aperture area of 0-90-135 is 4.225×10^5^ um^2^, while 0-90-45 can be regarded as the result of rotating 45^o^ on the basis of 0-90, and the original aperture is divided into four triangles with a base of 400 um and a height of 356.25 um, with a theoretical aperture area of 2.85×10^5^ um^2^. Specific fiber sizes, spacings, and aperture diameters may deviate from the design parameters at the time of printing, but the scaffolding parameters, but the scaffolds were all made in the same batch, which did not affect the trend of scaffold structure change and experimental results. In addition, the porosity of the scaffolds is more than 70%, which we obtained through the porosity test experiment.

How did you monitor the Ca and P release ?

Dear reviewer, the release of Ca and P ions was not monitored in this study, and we only extrapolated the reasons for HA regulation of BMSCs and HUVES behavior based on existing studies such as Yifan Niu, Carvalho, MS et al[3, 4].

[3] Y. Niu, L. Chen, T. Wu, Recent Advances in Bioengineering Bone Revascularization Based on Composite Materials Comprising Hydroxyapatite, International Journal of Molecular Sciences 24(15) (2023).

[4] M.S. Carvalho, J.M.S. Cabral, C.L. da Silva, D. Vashishth, Bone Matrix Non-Collagenous Proteins in Tissue Engineering: Creating New Bone by Mimicking the Extracellular Matrix, Polymers 13(7) (2021).

How can we interpret this “Furthermore, in scaffolds with smaller pore sizes, the preference is for cell growth rather than extracellular matrix secretion” – smaller than which value? There are many sentences which are unclear and not at all defined well – not clearly quantifiable.
Dear reviewer, our description is not rigorous, based on the results of other people's studies arbitrarily came to this conclusion, has been referenced to supplement, thank you for your correction. Sio-Mei Lien et al. found that pore sizes of 50-150 μm were suitable for cell proliferation, while cells with the largest pore sizes (250-500 μm) were more inclined to secrete extracellular matrix[4]. In our another study, compared to 800um, scaffolds with a pore size of 500um significantly promoted BMSCs differentiation[5].

[4] M.S. Carvalho, J.M.S. Cabral, C.L. da Silva, D. Vashishth, Bone Matrix Non-Collagenous Proteins in Tissue Engineering: Creating New Bone by Mimicking the Extracellular Matrix, Polymers 13(7) (2021).

[5] W. Huang, S. Huang, X. Linghu, W.-C. Chen, Y. Wang, J. Li, H. Yin, H. Zhang, W. Xu, Q. Wa, 3D-Printed mesoporous bioglass/ polycaprolactone scaffolds induce macrophage polarization toward M2 phenotype and immunomodulates osteogenic differentiation of BMSCs, 10(5) (2024).

Minor English polishing is still needed. For instance, instead of bio ceramics it should be bioceramics; instead of “to free more calcium and phosphorus components to help bone mineralisation” it is better to use “to release more calcium and phosphorus components to help bone mineralisation”; why you added “/” in “/A study by Barbara Ostrowska et al15” …Dear reviewer, we have revised it.
